# New tools for the investigation of muscle fiber-type spatial distributions across histological sections

**DOI:** 10.1186/s13395-023-00316-0

**Published:** 2023-04-22

**Authors:** Anna K. Redmond, Tilman M. Davies, Matthew R. Schofield, Philip W. Sheard

**Affiliations:** 1 Department of Mathematics & Statistics, University of Otago, Dunedin, 9016 New Zealand; 2 Department of Physiology, University of Otago, Dunedin, 9016 New Zealand

**Keywords:** Skeletal muscle, Fiber type, Clumping, Aging, Neuromuscular disease, Pattern, Spatial distribution, Statistical methods

## Abstract

**Background:**

The functional and metabolic properties of skeletal muscles are partly a function of the spatial arrangement of fibers across the muscle belly. Many muscles feature a non-uniform spatial pattern of fiber types, and alterations to the arrangement can reflect age or disease and correlate with changes in muscle mass and strength. Despite the significance of this event, descriptions of spatial fiber-type distributions across a muscle section are mainly provided qualitatively, by eye. Whilst several quantitative methods have been proposed, difficulties in implementation have meant that robust statistical analysis of fiber type distributions has not yielded new insight into the biological processes that drive the age- or disease-related changes in fiber type distributions.

**Methods:**

We review currently available approaches for analysis of data reporting fast/slow fiber type distributions on muscle sections before proposing a new method based on a generalized additive model. We compare current approaches with our new method by analysis of sections of three mouse soleus muscles that exhibit visibly different spatial fiber patterns, and we also apply our model to a dataset representing the fiber type proportions and distributions of the mouse tibialis anterior.

**Results:**

We highlight how current methods can lead to differing interpretations when applied to the same dataset and demonstrate how our new method is the first to permit location-based estimation of fiber-type probabilities, in turn enabling useful graphical representation.

**Conclusions:**

We present an open-access online application that implements current methods as well as our new method and which aids the interpretation of a variety of statistical tools for the spatial analysis of muscle fiber distributions.

**Supplementary Information:**

The online version contains supplementary material available at 10.1186/s13395-023-00316-0.

## Background

Skeletal muscles are complex structures with substantial location- and usage-related variability in fiber number, diameter, length, and arrangement [1]. Fibers are also classified by their contractile and metabolic properties into one of four primary fiber type groupings, one of which is classified as “slow” whilst the others are “fast” subtypes [2], though all fiber subtypes are not necessarily present in all muscles [3]. Despite their structural and functional complexity, the arrangement of fibers within any given skeletal muscle is strikingly similar amongst the normal individuals of any species [3], and this arrangement is thought to arise as a consequence of signals received from the motor nerve terminal [2].

Muscle is “plastic,” in the sense that any fiber’s functional and metabolic properties may change in response to altered usage pattern or innervation status, as has been demonstrated many times by experimental interventions involving cross-reinnervation or externally imposed usage patterns [4]. Alteration of fiber type profiles also changes with age and with neuromuscular pathologies [5, 6], likely reflecting an age- or disease-related change in usage pattern or innervation status; these phenotypic changes are often accompanied by a reduction in muscle mass and strength and are characterized by the emergence of apparent fiber type clusters in which the grouping and proportion of slower fiber types increases [5].

The strong control over muscle fiber type exerted by the motor nerve is a compelling reason to use the spatial distribution of fiber types as an indicator of pathological processes integral to neuromuscular aging or disease. Our understanding of these processes would be greatly enhanced if we were able to identify whether they followed any particular pattern or exhibited objectively identifiable spatial behaviors. Many muscles have stereotypical distributions of fast and slow fibers throughout [3], so a change in this pattern has the potential to provide new insight into the primary causes of the change. For example, an age-related faster-to-slower fiber type switch and apparent clustering might reflect a process in which motor neurons projecting to fast fibers die earlier in normal aging than those projecting to slow fibers [6]. In addition, if the fiber type switching process occurred across the muscle with a non-random pattern, it might indicate that the death of motor neurons is related, at least in part, to the location of their terminals in the periphery. A similar proposal could be raised to explain any consistent emergent pattern change in neuromuscular disease. To this end, we need accurate quantitative methods for describing the distribution of fiber types across muscle sections.

In studies that feature microscopic imaging of muscle sections, interpretations as to the spatial configurations of fast and slow fibers are often based on subjective visual inspection [7], an approach with obvious drawbacks. Statistical approaches provide more objective options to undertake research addressing basic biological principles, including how skeletal muscle changes as we age, and how neuromuscular pathologies manifest and progress. Continuing with an age-related example, a better understanding of the cellular changes that drive neuromuscular deterioration in normal aging will support the development of evidence-based therapies to slow the process and thereby to help people retain their independence further into their old age.

The problems associated with subjective visual appraisals of muscle fiber-type distributions and the appeal of targeted statistical methods were raised 40 years ago [8] and subsequently included a consideration of the geometric methods used in processing the data sets [9, 10], novel statistical tests designed to seek evidence against a null hypothesis of a completely random distribution [11–13], and moving toward the use of formal modeling techniques to more precisely describe the spatial configurations of dichotomized muscle fiber data [14–16]. There are also more recent examples of approaches to quantify spatial relationships in muscle fiber cross-sections [17–20]. Despite the attempts of several authors to raise the issue for discussion and propose their own analytical solutions, the specialized nature of the statistical methodology has meant that routine applications of these methods are few.

The objectives of this work are threefold. First, we review some of the historically proposed methods referenced above, omitting technical detail in favor of a focus on application and interpretation. Second, we suggest and demonstrate the application of modern statistical techniques that have not been previously considered in this context. These approaches are capable of offering greater insight into the spatial structure of binary fiber-type data and can be easily extended to cases where there are more than two fiber types—thereby aiming to highlight the utility with which readily available statistical tools can serve relevant research pursuits. Finally, we seek to improve accessibility to these analytical techniques by introducing a freely available web-based application in which the user can upload their own data to obtain summaries from a variety of statistical approaches via a point-and-click interface.

## Methods

### Motivating examples and data processing

**Fig. 1:**
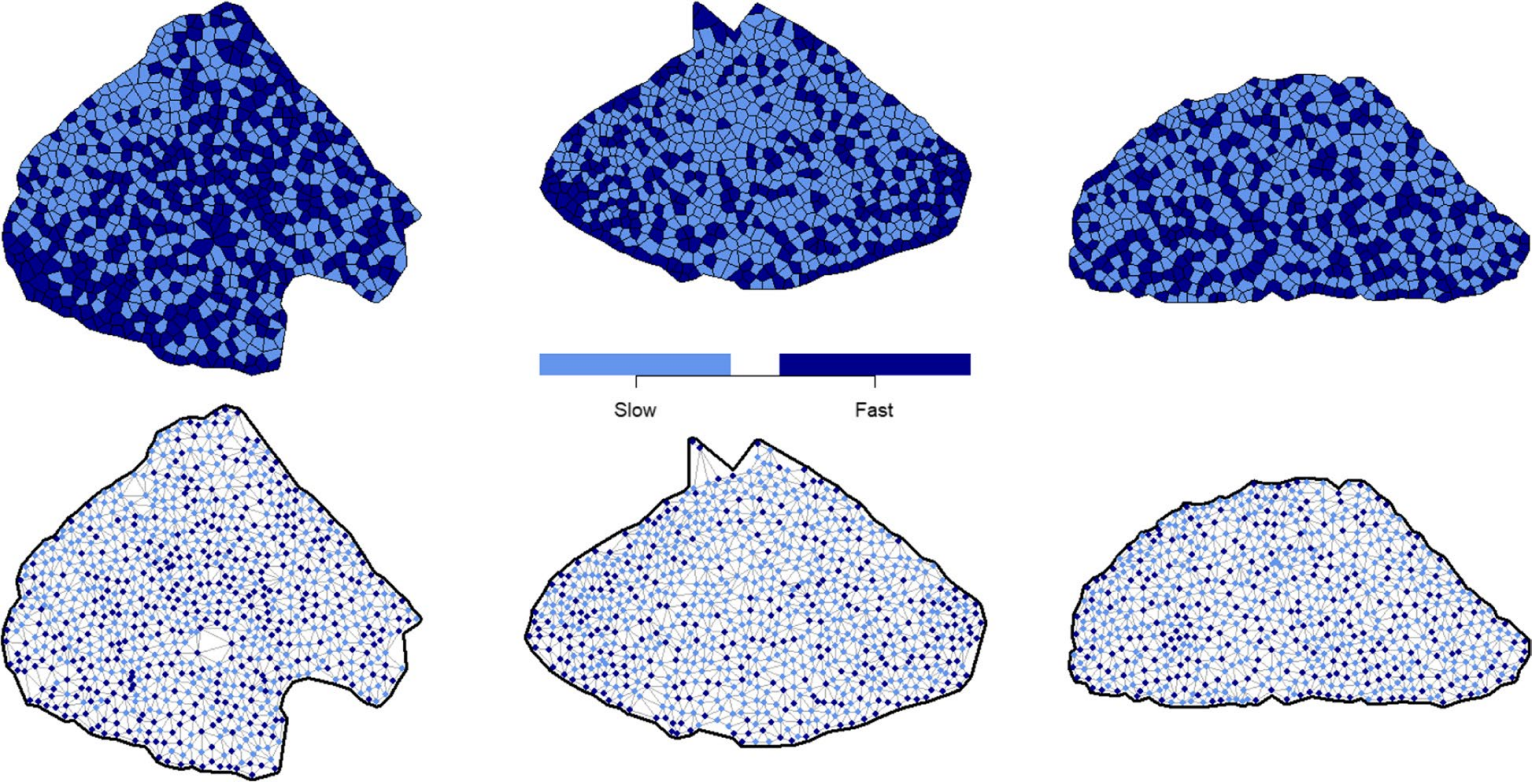
Digitized, geometrically treated versions of the three example sections, shown as tessellations (top row) or triangulations (bottom row)

To facilitate the ensuing discussion, we consider three example data sets (Fig. 1). These specimens represent cross-sections of the soleus muscles of three different mice, sections having been histochemically stained to identify fast fibers. The mouse soleus comprises primarily a mix of type I and type IIA fibers, so treatment of the data as a set of binary indicators is appropriate in this case. A casual visual inspection of the leftmost muscle suggests an area in the top left of the section with mainly slow fibers, and the bottom left has mainly fast fibers, but the rest of the section has no obvious patterns or clustering. The center example appears to have a higher proportion of slow fibers near the center of the section, with the fast fibers mostly grouped together around the edges. The rightmost specimen has an even distribution of both types across the whole section, with very little visually obvious grouping together of fibers of the same type.

In experimental settings, stained muscle sections are typically photographed through a microscope and digitally processed. The data required for statistical analysis are two-dimensional coordinates—$\left(x,y\right)$—that provide the centroid of each fiber, along with the classified fiber type. Using this information, standard geometric methods are used to derive a polygon approximating the overall shape of the muscle and to identify spatial neighbors of each fiber—yielding a “neighbor network”. Figure 1 shows two different ways of visualizing the result using our three example sections: a tessellation and a triangulation. The latter is preferable if explicit visualization of the neighbor network is desired. Additional details on the geometric techniques mentioned here appear in the supplementary materials.

### Summary- and test-based methods

Here, we briefly review three existing methods developed as simple summaries and hypothesis tests for binary (i.e., fast/slow) muscle fiber data. While fast and convenient, they are focused on answering “global” questions related to type-specific randomness (or lack thereof) across the entire sample.

#### Mean cluster size

One of the first targeted statistical tests involved the analysis of the size of clusters of like-type fibers. Howel and Brunsdon [11] considered a null hypothesis of random scattering of a chosen fiber type across the muscle section. They implemented an iterative search algorithm to establish the *mean cluster size* (where “cluster” is simply defined as a contiguous group of like-type fibers) of the less prevalent fiber type. This statistic is compared against a 95% *permutation envelope,* empirically derived assuming randomness. If the mean cluster size for the fiber type of interest is abnormally large or small, this suggests that fibers tend to “attract” or “repel,” respectively, others of the same type. Exceedance by the test statistic of the permutation envelope on either side suggests statistically significant evidence against randomness. This approach is assumed reliable when the less prevalent fiber type makes up less than 30% of the fibers in the sample, which may not always be the case in practice.

#### Unlike neighbor pairs

A hypothesis test of a similar nature was developed by Venema [13]. He suggested using the number of unlike neighbor pairs as a test statistic. The rationale is that a section where the arrangement of the two fiber types is random should have a mix of pairs of neighbors of both slow, both fast, and one of each type. Strong like-type clustering in the section would result in fibers tending to appear beside other fibers of the same type, so there would be more pairs of neighboring fibers of the same type and hence fewer unlike neighbor pairs.

Venema [13] demonstrated the calculation of the mean and variance of the number of unlike neighbor pairs under a null hypothesis of randomness. Using the observed number of unlike neighbor pairs to this distribution, a test statistic can be found that is negative when the sample specimen exhibits like-type clustering and positive for like-type segregation. A *p* value can then be calculated to determine the statistical significance of any departure from randomness.

#### Abnormally grouped fibers

A more recent approach [18] involves calculating the proportion of “abnormally grouped” slow fibers as a descriptive statistic. Under the assumption of a random distribution, for each slow fiber, we calculate the mean and standard deviation for the number of other slow-type neighbors. If there are at least two neighboring fibers in a cluster where the number of other slow fibers neighboring each of these exceeds one standard deviation above the mean, all fibers in that cluster are subjectively classified as abnormally grouped.

Unlike the other methods described above, these proportions do not represent a formal statistical test and hence cannot be usefully interpreted in terms of whether the distribution is random, but are instead designed to be compared between different categories of sections with similar overall type-specific proportions in order to summarize differences in like-type behavior between groups. The authors of this approach observed heightened slow fiber grouping in older human muscles (60–75 years) compared to younger muscles (20–35 years). Unfortunately, the statistic is highly sensitive to the overall proportion of slow fibers since a higher proportion of slow fibers naturally leads to larger clusters. As such, only muscles with similar proportions of slow fibers should be compared using this statistic. See the supplementary material for further elucidation.

### Model- and smoothing-based methods

Useful though they are, test-based methods like those discussed above have limited inferential scope. For example, hypothesis tests are not able to provide readily interpretable measures of strength or magnitude of any like-type behavior (merely answering a “yes/no” question of spatial randomness), nor are they able to offer insight into specific spatial structure (i.e., in terms of *where* in a given section like-type fibers might be more likely to cluster). For these types of questions, we need more sophisticated methods. Fortunately, there are statistical tools that lend themselves well to such analyses and we will review them here in the context of binary muscle fiber data.

#### Binary Markov random field

Venema [15, 16] was the first to suggest a model-based approach to the analysis of binary-valued muscle fiber data. His main proposal was to consider the two types of fibers as arising from the simplest form of a *binary Markov random field* (BMRF). Suppose the $n$ fibers in a particular muscle section are defined as $\mathbf{z}=\{{z}_{1},\dots ,{z}_{n}\}$, where ${z}_{i}$ is $-1$ if the $i$ th fiber is slow, or $+1$ if it is fast. These are treated as a realization of a corresponding random variable $\mathbf{Z}$. The BMRF is used to model the probabilities of different combinations of $\mathbf{z}$; expressed as a function of the fiber values with respect to the neighbor network:

1 $${\text{Pr}}\left(\mathbf{Z}=\mathbf{z}\right)\propto \mathrm{exp}\left\{\alpha \sum_{i}{z}_{i}+\beta \sum_{i\sim j}{z}_{i}{z}_{j}\right\}$$

Here, $\alpha$ and $\beta$ are fixed, and the notation $i\sim j$ indicates the second sum is taken only over each neighbor pair.

The key to understanding Eq. (1) is the interpretation of the parameters $\alpha$ and $\beta$. The former simply describes the balance between the numbers of slow and fast fibers—a value close to zero implies the counts are roughly equal; a negative value implies there are more slow than fast; a positive value yields more fast than slow. The interest lies typically in the value of the other parameter, $\beta$, which controls the behavior between neighbors. When $\beta$ is close to zero, this is equivalent to a “null” scenario where fast and slow types are randomly scattered throughout the sample. A negative value of $\beta$ implies fibers of the same type are less likely to be neighbors—that they are negatively correlated spatially—like-type *repulsion*. A positive value of $\beta$ dictates positive correlation—like-type *attraction*. Repulsion leads to a chequerboard effect, and attraction yields clusters of fibers of the same type. For details on the methods used to gain the parameter estimates of $\alpha$ and $\beta$ in practice, see Venema [16] or Davies et al. [17]. The estimated value of $\beta$ may be compared to a numerically derived *permutation envelope,* which essentially acts as a benchmark for establishing whether it is significantly different from zero.

The BMRF approach thus has several advantages over the simpler tests. Not only may we ascertain the presence or absence of non-randomness in the spatial arrangement of the two fibers, but the *strength and direction* of any present like-type neighborly behavior may be formally quantified via the estimated value of $\beta$ (and this interpretation is valid for any overall proportion of slow versus fast fibers).

#### Generalized additive model

One thing the simple BMRF described above cannot do is inform on explicit spatial patterning in subregions of a given muscle cross-section; i.e., localize precisely *where* in a specimen we might observe features of interest. Rather than simply deducing whether or not a given section possesses like-type clustering, it may be of interest to model the *probability* of a chosen fiber type occurring and look at how this probability varies spatially across the section, especially in muscles where the typical distribution of fibers is non-uniform [3].

The probability ${p}_{i}$ that a given fiber $i$ at coordinate $\left({x}_{i},{y}_{i}\right)$ is fast can be modeled to be a smooth function of its position in the muscle section through the use of a *generalized additive model* (GAM) [21]. To our knowledge, GAMs have not previously been used to model spatial patterning in muscle fiber position data. A GAM is an extension of a generalized linear model (GLM) that allows the relationship between the predictors and the response to be smooth but non-linear. In our case, we model the log odds of a given fiber being fast via a logistic GAM, expressed as follows:

2 $$\mathrm{log}\left(\frac{{p}_{i}}{1-{p}_{i}}\right)={\beta }_{0}+{\beta }_{1}{x}_{i}+{\beta }_{2}{y}_{i}+\sum_{k=3}^{K}{\beta }_{k}{b}_{k}\left({x}_{i},{y}_{i}\right)$$

This equation is very similar to the form of a logistic regression model but with terms added to allow for greater flexibility in the resulting trend surface. The functions ${b}_{3},\dots {b}_{K}$ are non-linear and are chosen to allow the resulting function to closely match the patterns in the data. A common choice is a *thin plate spline regression basis* [22]. Estimation of the $\beta$ parameters (note that these are completely different to the $\beta$ used in the BMRF method) relies on a “penalty” parameter we call $\lambda$, which controls the “smoothness” of the overall result. There are data-driven techniques to choose an optimal $\lambda$. For more details, see the supplement and the work by Wood [21, 22].

Once we have fitted a logistic GAM to a muscle section data set, we can visualize the trend in fiber type probabilities across the section, revealing potential features of interest. Unlike the previous methods outlined above, fitting a GAM does not require explicit knowledge of the neighborhood structure. However, the natural effect of smoothing means the probability for a given fiber will be influenced mainly by nearby fibers.

#### Multinomial GAM

A weakness of the methods described is that they are designed for data that are binary. It is in general not straightforward to extend the methodology to cases where there are more than two classes, for example, if we were to further classify type II fibers into subtypes IIa and IIb. The exception is the GAM which can easily be extended to allow for three (or more) fiber types. A multinomial GAM [21] has a categorical response variable with a probability associated with each category. These probabilities are described in a similar way to Eq. (2) and use the same functions ${b}_{3},\dots {b}_{K}$ from the thin plate regression spline basis. For three fiber types (I, IIa, and IIb), we construct two surfaces each with its own set of $\beta$ parameters and smoothing parameter $\lambda$. Because the probabilities are required to sum to 1 for each fiber, these two surfaces are used to determine probabilities for each of the three types.

### Implementation

All of the above methods can be implemented in standard statistical software such as the R language [23]. We have developed a browser-based application [24] to showcase these techniques and produce relevant plots and conclusions for a supplied dataset. The user needs to supply a CSV file with columns for the *x* coordinate, *y* coordinate, and fiber type for each fiber. The app will pre-process the data and produce plots such as those in Fig. 1. Available options in the app include the mean cluster size and number of unlike neighbor pairs tests, as well as the “proportion abnormally grouped” statistic. It can also estimate and interpret the parameters for the BMRF model. For the logistic GAM model, the app calculates and plots the probabilities of each fiber being fast contracting/type II. If desired, the user can manually vary the value of the smoothing parameter $\lambda$ from a standard default and visualize the resulting probability surface. The multinomial GAM is also available if the supplied data file has three fiber types, and the app calculates and plots the probability surfaces for each type. The application is accessible at https://www.stats.otago.ac.nz/software/muscles/ (uploaded data is not visible to any party beyond the user themselves and is permanently erased upon browser close or refresh).

## Results

### Summary- and test-based methods

Table 1 shows the results of the three summary- and test-based methods applied to the same three example sections. The data for these three specimens are provided alongside the online supplement to this article in a form ready to be used by the web application mentioned above.

**Table 1:** Summary- and test-based results for the three example sections

	**Section**		
	**1**	**2**	**3**
Mean cluster size	11.6	5	4.4
Envelope	(10.7, 21.4)	(4, 5.8)	(4.5, 6.9)
Conclusion/Evidence (mean cluster size test)	Random	Random	Like-type repulsion
Expected unlike neighbor pairs	1187.1	1037.4	957.3
Observed unlike neighbor pairs	1181	955	1021
*p*-value	0.8177	$2\times {10}^{-4}$	0.0033
Conclusion/Evidence (unlike neighbor pairs test)	Randomness	Like-type attraction	Like-type repulsion
% abnormally grouped slow fibers	85.7%	96%	95.6%
% abnormally grouped fast fibers	65.2%	55.3%	28.4%

The results for the mean cluster size test show the mean cluster size in each sample, along with an envelope in which the statistic would be expected to fall if the fiber types were allocated randomly. Note that in all three cases, the rarer fiber type count exceeds the guideline of 30%, and so results should be interpreted with caution. For the first two sections, the mean cluster size is within the range expected from a random fiber distribution so there is no evidence of like-type clustering. The mean cluster size for the third section is below the envelope, indicating the clusters are smaller than would be expected of a random fiber distribution, so there is evidence of like-type repulsion.

For the unlike neighbor pairs test, Table 1 shows the expected number of unlike neighbor pairs, based on the assumption of a random distribution, as well as the number observed in the data, with an associated *p* value to indicate whether the results yield evidence of non-randomness. The first section has 1181 unlike neighbor pairs, which is only slightly less than the expected value, so this is not a significant departure from randomness. The number of unlike neighbor pairs for the second and third specimens (955 and 1021, respectively) do give significant *p* values, suggesting evidence of like-type attraction for the second section and like-type repulsion for the third.

We also looked at the percentage of abnormally grouped fibers. The first section has 51% slow fibers, the majority of which (85.7%) belong to either of two very large “abnormal” clusters. Similarly, both of the other two sections have most (96% and 95.6%, respectively) of their slow fibers in one contiguous group and all are subsequently classified as abnormally grouped. When the analysis focuses on the (less prevalent) fast fibers the percentages found as abnormally grouped are lower, with the first and second specimens having more “abnormal” clustering than the third. These differences in results based solely on whether we focus on either slow or fast fibers highlight the caution required when comparing these statistics between specimens with different overall fiber-type proportions.

### Model- and smoothing-based methods

First, the BMRF was fitted to our three example sections, and the results are reported in Table 2.

**Table 2:** Results of the BMRF fitted to the three example sections

	**Section**		
	**1**	**2**	**3**
Estimate for $\alpha$	− 0.0165	− 0.177	− 0.348
Estimate for $\beta$	0.00349	0.0636	− 0.0902
Envelope for $\beta =0$	(− 0.0363, 0.0295)	(− 0.046, 0.0414)	(− 0.0423, 0.0445)
Conclusion/Evidence	Random	Like-type attraction	Like-type repulsion

All three sections have a negative estimate for $\alpha$, confirming slow fibers outnumber fast fibers. After accounting for the overall proportions of each fiber type, the first two examples have positive estimates for $\beta$, suggesting like-type attraction. For the first specimen, this value is very close to zero and within the envelope of values expected from a random distribution, hence does not indicate a departure from randomness. For the second section, the estimate for $\beta$ is outside of the envelope; significant evidence that the distribution of fibers is non-random. The estimate is also outside of the envelope for the third section, but this time it is negative, indicating significant evidence of like-type repulsion.

**Fig. 2:**
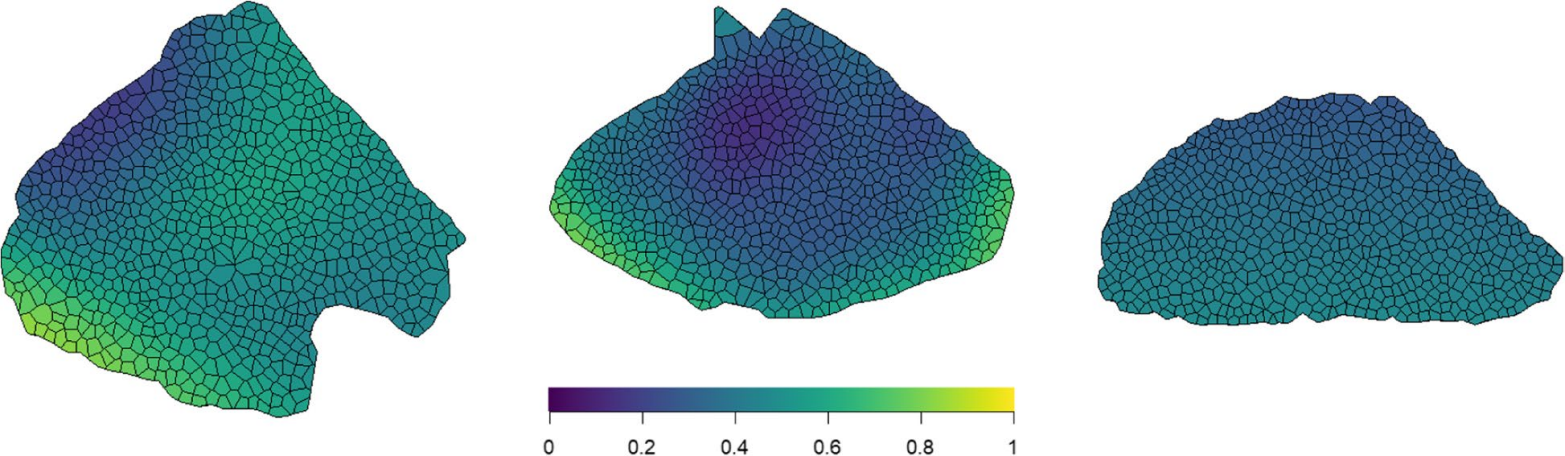
Probability of a fiber showing a fast phenotype estimated from a logistic GAM

Next, we fitted the (binary) logistic GAM as expressed by Eq. (2) to the three example sections. These permit the calculation of the conditional probability of a fiber at any given $\left(x,y\right)$ location being fast; corresponding plots are shown in Fig. 2. Rather than measuring clustering behavior over the muscle as a whole, these plots show how the probability of a particular fiber type is estimated to fluctuate across the specimen.

The first plot shows a similar pattern as is observed from the fiber-type plots. For example, there is a prominent region in the top left of the sample where the probability of a fiber being fast is low. The second image suggests the probability of a fiber being fast is highest near the edges of the section and lowest near the center of the section. The third image shows the section does not have a strong pattern in the fiber types, as the probability of a fiber being fast is fairly constant across the whole section, roughly between 0.3 and 0.5. Given that many muscles have a non-uniform distribution of fiber types across the muscle [3], these plots can facilitate assessments of change in said distribution as a function of biologically important variables such as age or disease.

**Fig. 3:**
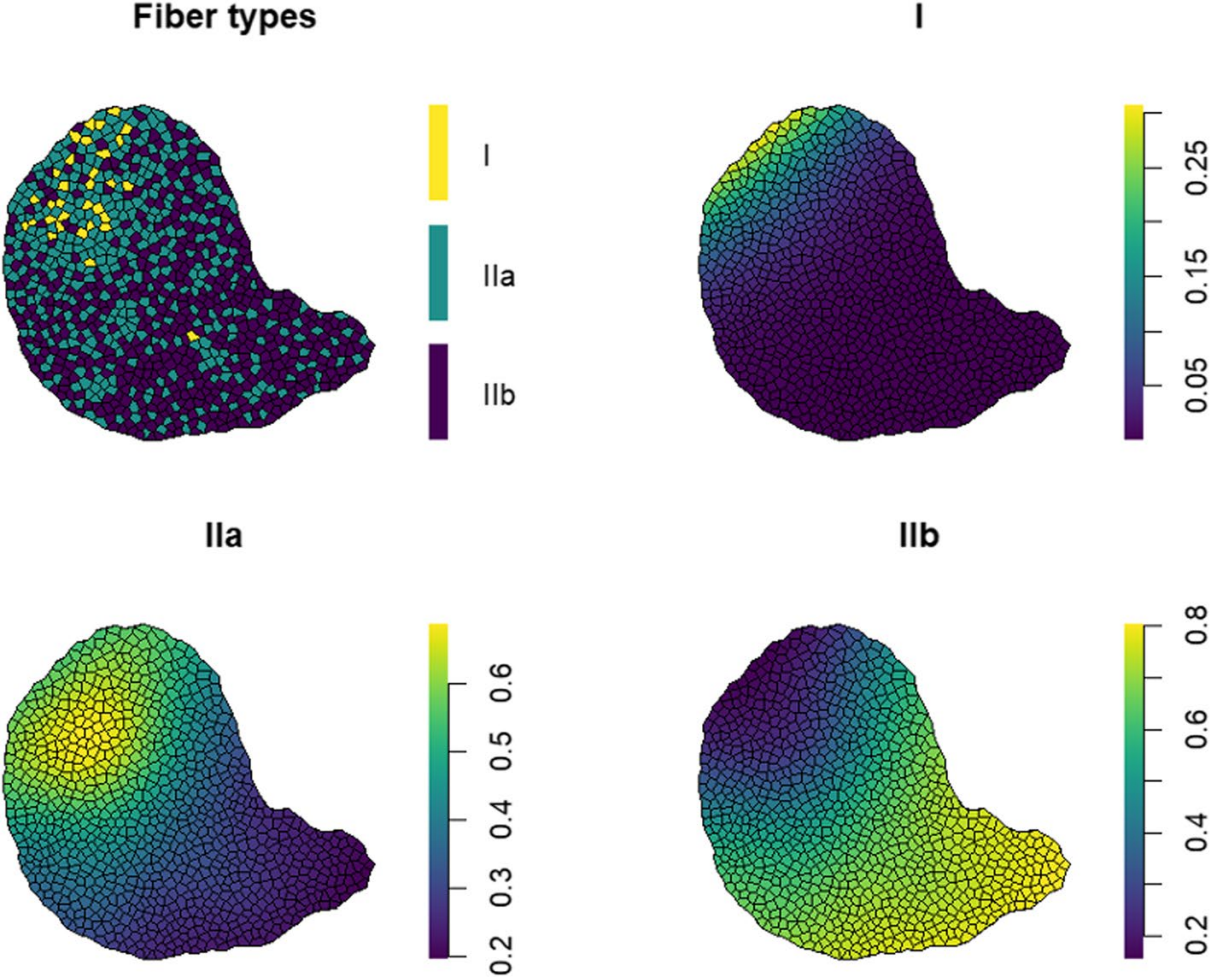
Fiber types (top left) and probability surfaces for types I (top right), IIa (bottom left), and IIb (bottom right) estimated using a multinomial GAM

To illustrate the multinomial GAM, we introduce a data set simulated to mimic the distribution of three fiber types (I, IIa, and IIb) in a rodent tibialis anterior muscle (Fig. 3). After fitting a multinomial GAM, Fig. 3 shows the type-specific probabilities for each fiber. There are few type I fibers, so the probability of being type I is low across the section, with a maximum value of about 0.3 in the top left part of the section (deep in the muscle). The probabilities of being type IIa and IIb both range from around 0.2 to 0.8, with a higher probability of being type IIa in deep regions (upper left of section) and a higher probability of being type IIb in superficial areas (lower right of the section).

### A group-based comparison

**Fig. 4:**
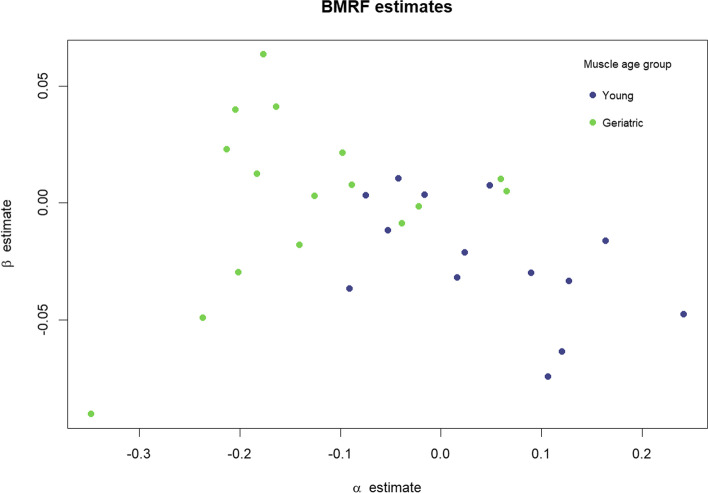
BMRF parameter estimates for mouse soleus muscles from two age groups

Certain tools can assist with important group-based comparisons frequently encountered in practical applications, for instance, when examining differences between age- or disease-related sections. To illustrate a simple way in which we might do so using existing methodologies, we consider a larger group of 30 mouse soleus muscles, split into two age subgroups—14 from young mice and 16 from older animals (the three sections in Fig. 1 used as running examples were taken from this collection). We fit the BMRF model to each specimen, obtaining estimates of the parameters $\alpha$ and $\beta$ for each; results are illustrated with a scatterplot in Fig. 4. Here, we note the geriatric muscles tend to have lower values for $\alpha$ and higher values for $\beta$ than the younger muscles, suggesting that (a) they contain a lower proportion of fast fibers and (b) a higher tendency of like-type spatial grouping when compared to the group of young muscles.

**Fig. 5:**
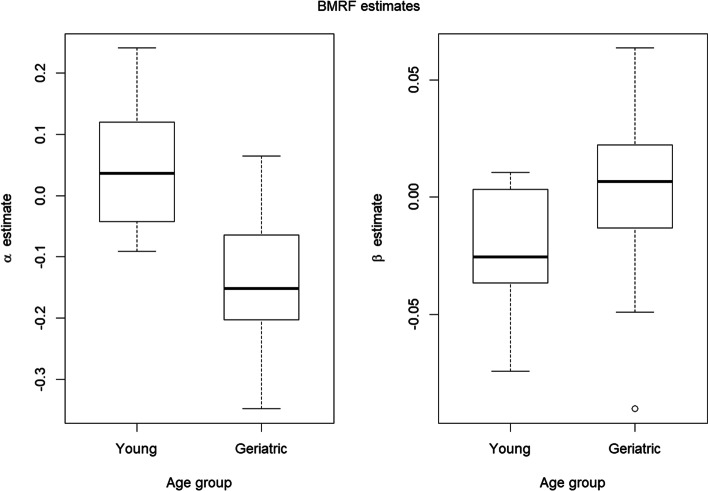
Boxplots comparing the estimates for the two parameters of the BMRF model between young and geriatric mouse soleus muscles

Boxplots in Fig. 5 show the distinctions in the marginal distributions of the two parameters. Given these, it is unsurprising that a simple *t* test gives evidence that on average, the estimates for the $\alpha$ parameters are significantly lower for the muscles in the older age group (*p* value $5.8\times {10}^{-5}$), and the estimates for the $\beta$ parameters are significantly higher for the geriatric muscles (*p* value $0.031$). The Mann–Whitney *U* test, a non-parametric test with less restrictive assumptions typically used in the case of small sample sizes, offers the same conclusions for both $\alpha$ (*p* value $1.2\times {10}^{-4}$) and $\beta$ (*p* value $0.017$).

## Discussion

Muscle fiber type initially arises during early development as a function of the specific lineages of cells contributing to muscle fiber formation [25–27], but the mature fiber-type distribution emerges as a consequence of interaction with the nerve, including usage pattern and molecular signaling [2]. Changes to the number or spatial distribution of fiber types within the muscle are therefore indicative of an alteration in muscle usage pattern or innervation status. Our understanding of both normal aging and neuromuscular disease processes would be improved if we understood how and why such alterations in usage occurred, particularly since the consequences of these changes are typically weakness and loss of muscle mass—a topic of importance recognized by a number of authors to date [28]. An essential part of this pursuit is to have available a range of statistical tools to offer a more objective appraisal of spatial patterns in muscle sections. To this end, we have highlighted existing methods and proposed new ways to quantify muscle fiber-type distributions. The simple summary-based methods are designed to detect like-type attraction or repulsion in a given section. The two model-based methods allow for richer statistical inferences to be made about the fiber type distribution across a section. The BMRF model can quantify the level of like-type attraction or repulsion between fibers over the section as a whole after adjustment for overall fiber-type proportions. The logistic GAM can provide an insight into any patterns in the type distribution across the muscle section and allows us to visualize the spatial trends. This might be particularly valuable when asking whether a change in a major spatial trend has occurred as a result of aging or disease. Additionally, we have developed a web-based tool to facilitate ready access to all described methods by non-statisticians.

The summary-based approaches and BMRF model are reliant on the binary demarcation of fiber type. That is, the processes are applied to images of muscle sections in which (immuno-) histochemical techniques have marked the fibers as either fast or slow. In most cases, this is an oversimplification of the true status of the fast fiber subsets, since not only can the fast subcategory be further subdivided (i.e., types IIa, IIb, IIx), but individual fibers might also display the features of more than one category at any one time [29]. Such “hybrid” fibers are typically thought to have been sampled at a time when fiber-type transformation was occurring due to the recent imposition of an altered usage pattern. Unlike the other methods, the logistic GAM can be easily extended to a multinomial model to allow for three or more fiber types, making this a more flexible approach. However, this still requires a choice of how many types to allow and each fiber must then be classified into one category. The digitization of the (immuno)histochemical staining process gives continuous color values to support the fiber-typing criteria and analyzing these directly instead would avoid the need to make such threshold-based categorizations, as well as allowing hybrid fibers to have values between the two main fiber types. We are currently investigating more general statistical methods to better cope with such data sets.

A limitation of all existing methods is that they are specific to a given muscle section. While it is entirely possible to conduct post hoc hypothesis tests between groups for some of the simpler measures (as we have demonstrated with the BMRF fitted to young and geriatric sections), it is preferable to develop structured models that explicitly build in support for group-specific effects. We are currently undertaking further research to develop new, statistically rigorous methodologies to formalize between-group comparisons.

## Conclusions

The currently available methods for the analysis of fiber type distributions on histological skeletal muscle sections deliver variable results when applied to the same specimen and are limited in their ability to support analysis of distributions across the entire section. Furthermore, current models have had little uptake in practice due to difficulties associated with implementation. In addition to summarizing the previous methodologies, we have described a new approach via a logistic generalized additive model; capable of providing an insight into the fiber type distribution across the section as a whole and easily extended to describe the distributions of three or more fiber types. We provide access to a freely available web-based application that allows users to process their own datasets using all methods described herein. This simple-to-use software gives the community access to a powerful suite of analytical tools that provides a robust interpretation of spatial data, thereby removing a significant barrier to statistical analysis of changes in fiber type distributions in systems affected by age or disease.

## Supplementary Information

**Additional file 1. **New tools for the investigation of muscle fiber-type spatial distributions across histological sections. **Figure S1.** Proportions of abnormally grouped type I fibers for simulated data with randomly allocated fiber types. **Figure S2.** Plots of fiber types for 30 mouse soleus muscles, 14 young and 16 geriatric.

**Additional file 2.**

## Data Availability

The datasets used in this study are available as supplementary information files. The software developed and described is freely accessible at https://www.stats.otago.ac.nz/software/muscles/.

## Abbreviations

GAM: Generalized additive model
GLM: Generalized linear model
BMRF: Binary Markov random field
