# Supplementary material for: New tools for the investigation of muscle fiber-type spatial distributions across histological sections
Source: Skelet Muscle. 2023 Apr 22;13:7. doi: 10.1186/s13395-023-00316-0 (PMC10122286; doi:10.1186/s13395-023-00316-0)
Supplement: Supplementary file 1 — Additional file 1. New tools for the investigation of muscle fiber-type spatial distributions across histological sections. Figure S1. Proportions of abnormally grouped type I fibers for simulated data with randomly allocated fiber types. Figure S2. Plots of fiber types for 30 mouse soleus muscles, 14 young and 16 geriatric. [file 13395_2023_316_MOESM1_ESM.docx]

SUPPLEMENTARY MATERIAL: New tools for the investigation of muscle fiber-type spatial distributions across histological sections

Anna K. Redmond, Tilman M. Davies, Matthew R. Schofield, Philip W. Sheard

UNIVERSITY OF OTAGO, NEW ZEALAND

1. *Geometric processing methods*

Here we comment specifically on the geometric methods used to prepare a muscle section for statistical analysis. From the $\left( x,y \right)$ coordinates of each fiber, we need to find the shape of the muscle boundary and determine the neighbors of each fiber.

To compute the shape of the muscle boundary, we use the $\alpha$*-shape^1^*. This is closely related to the *convex hull* but is more flexible, making it appropriate for use when the shape displays ‘inward’ curves or concavity at the boundaries. Functions to compute the $\alpha$-shape are available in existing statistical software and have been successfully used for muscle fiber data analysis^2^.

Many of the analyses we describe also require identifying the neighbors of each fiber in the muscle. For this we use the *Voronoi tessellation* and closely related *Delaunay triangulation^3^*. The Voronoi tessellation divides the muscle shape into a tile for each fiber, where each tile delineates the portion of 2D space that is closer to the corresponding fiber than any of the other fibers in the muscle. This tessellation is truncated to the $\alpha$*-shape* of the muscle. Finally, the Delaunay triangulation is obtained by connecting two fibers as neighbors when their corresponding Voronoi tiles share an edge.

The techniques as described here are automatically executed when a suitable dataset is uploaded to the web-app accompanying the paper.

1. *Background and additional details on generalized additive models*

To motivate the GAM as defined in the paper, we can begin in more familiar territory. A standard logistic regression model is a natural approach to dealing with binary response data. One of the family of *generalized linear models*, logistic regressions express the probability of a specific outcome occurring, in the form of its log-odds or “logit”, in terms of a linear combination of predictor variables. Suppose we are interested in modelling the spatially varying probability, $p_{i}$, that a given fiber $i$ is fast, and let the spatial coordinates of the $i$th fiber be denoted with $x_{i}$ and $y_{i}$ on the horizontal and vertical axes respectively. A simple logistic regression would take the form

$$\log\left( \frac{p_{i}}{1-p_{i}} \right)=\beta_{0}+\beta_{1}x_{i}+\beta_{2}y_{i}.$$

( 1 )

This model is too rigid for our needs, however. While the above function clearly permits the probability of encountering a fast fiber to vary based on spatial position, we are limited to a linear relationship (on the logit scale) in the $x_{i}$ and $y_{i}$. In practical terms, this means the model will be incapable of capturing complex section-wide behavior in the fiber-types.

A *generalized additive model* (GAM)^4^ can afford the researcher more freedom in stipulating how the probability $p_{i}$ varies across the section. In general, it involves a linear combination of *functions* of the predictors, instead of a linear combination of the predictors themselves, as in logistic regression. A logistic GAM, the direct counterpart to logistic regression, has the same form as equation (1) but with additional terms imparting the desired flexibility:

$$\log\left( \frac{p_{i}}{1-p_{i}} \right)=\beta_{0}+\beta_{1}x_{i}+\beta_{2}y_{i}+\sum_{k=3}^{K} \beta_{k}b_{k}\left( x_{i},y_{i} \right)$$

( 2 )

In estimating the $\beta$ parameters, a ‘wiggliness’ penalty is calculated from the second derivatives in order to achieve a balance between the function being smooth and accurately modelling the pattern in the data. Because the functions $1, x_{i}$ and $y_{i}$ are completely smooth and have second derivatives of 0, the parameters $\beta_{0}\ldots\beta_{2}$ are not included in the penalty. The penalty is controlled by a smoothing parameter we call $\lambda$. If $\lambda$ is large, the estimates for the other parameters $\beta_{3},\ldots\beta_{K}$ will be shrunk close to zero and the resulting trend surface will be close to linear (a plane), and we essentially end up with a result no different to a standard logistic regression. If $\lambda=0$, there is no smoothing and the resulting function will be the one that most closely fits the observed data. We may use the observed data to select an appropriate value for $\lambda$, using a technique known as *generalized cross-validation*. Full technical details can be found in the text by Wood^4^.

The multinomial GAM is an extension to this model which can be used for data with more than two fiber types. For three fiber types, there are two surfaces of a similar form to equation (2), each with a set of $\beta$ parameters. Let $p_{i}^{\left[ IIa \right]}$ and $p_{i}^{\left[ IIb \right]}$ be the probabilities of fiber $i$, located at $(x_{i}, y_{i})$, being type IIa and IIb respectively, and type I is a reference category with probability $1-p_{i}^{\left[ IIa \right]}\boldsymbol{-}p_{i}^{\left[ IIb \right]}$. The surfaces modelling these probabilities are

$$\log\left( \frac{p_{i}^{\left[ IIa \right]}}{1-p_{i}^{\left[ IIa \right]}\boldsymbol{-}p_{i}^{\left[ IIb \right]}} \right)=\beta_{1,0}+\beta_{1,1}x_{i}+\beta_{1,2}y_{i}+\sum_{k=3}^{K} \beta_{1,k}b_{k}\left( x_{i},y_{i} \right)$$

$$\log\left( \frac{p_{i}^{\left[ IIb \right]}}{1-p_{i}^{\left[ IIa \right]}\boldsymbol{-}p_{i}^{\left[ IIb \right]}} \right)=\beta_{2,0}+\beta_{2,1}x_{i}+\beta_{2,2}y_{i}+\sum_{k=3}^{K} \beta_{2,k}b_{k}\left( x_{i},y_{i} \right)$$

( 3 )

and there is a smoothing parameter $\lambda$ for each set of $\beta$ parameters to penalize wiggliness in the same way as for the logistic GAM modelling two types. These equations can be used to find probability surfaces for each of the three fiber types.

1. *Cautionary details on use of the ‘proportion abnormally grouped’ statistic*

The abnormally grouped fibers method^5^ requires first calculating the mean and standard deviation for the number of slow fibers neighboring a given slow fiber.

For the $i$th fiber, the mean is $n_{i}p$ and the standard deviation is $\sqrt{n_{i}p\left( 1-p \right)}$, where $n_{i}$ is the number of neighbors of the $i$th fiber and $p$ is the overall proportion of slow fibers in the section. All fibers in a cluster of slow fibers are then classified as abnormally grouped if there are at least two contiguous fibers in the cluster where the number of slow fibers neighboring them exceeds one standard deviation above the mean.

While the proportion of abnormally grouped slow fibers would be expected to increase if like-type fibers tend to cluster together, the overall proportion of slow fibers also impacts this statistic. If there are a large number of slow fibers overall, the clusters tend to be larger. It then becomes likely that there are at least two fibers in a cluster surrounded by a lot of other slow fibers, resulting in the whole cluster being classified as abnormally grouped, even if the spatial distribution of types is completely random given the overall proportion of slow versus fast.

To demonstrate this phenomenon, Figure 1 shows results from this method applied to simulated data. For a given neighborhood structure, the fiber types for each fiber were randomly generated with varying probabilities of being slow. For each of the synthetic data sets, the proportion of abnormally grouped slow fibers was calculated. The plot shows that for very high proportions of type I fibers, there are no abnormally grouped fibers because the mean is so close to $n_{i}$ that it is impossible for the number of type I fibers neighboring the $i$th fiber to exceed one standard deviation above the mean. The rest of the plot shows a clear positive relationship with the proportion of abnormally grouped fibers, as the clusters are bigger, so it is more likely that at least one fiber in the cluster (and therefore also the rest) will be classifies as abnormally grouped. It is clear from this plot that it is not appropriate to compare the proportions of abnormally grouped type I fibers between two muscles with different overall proportions of type I fibers.


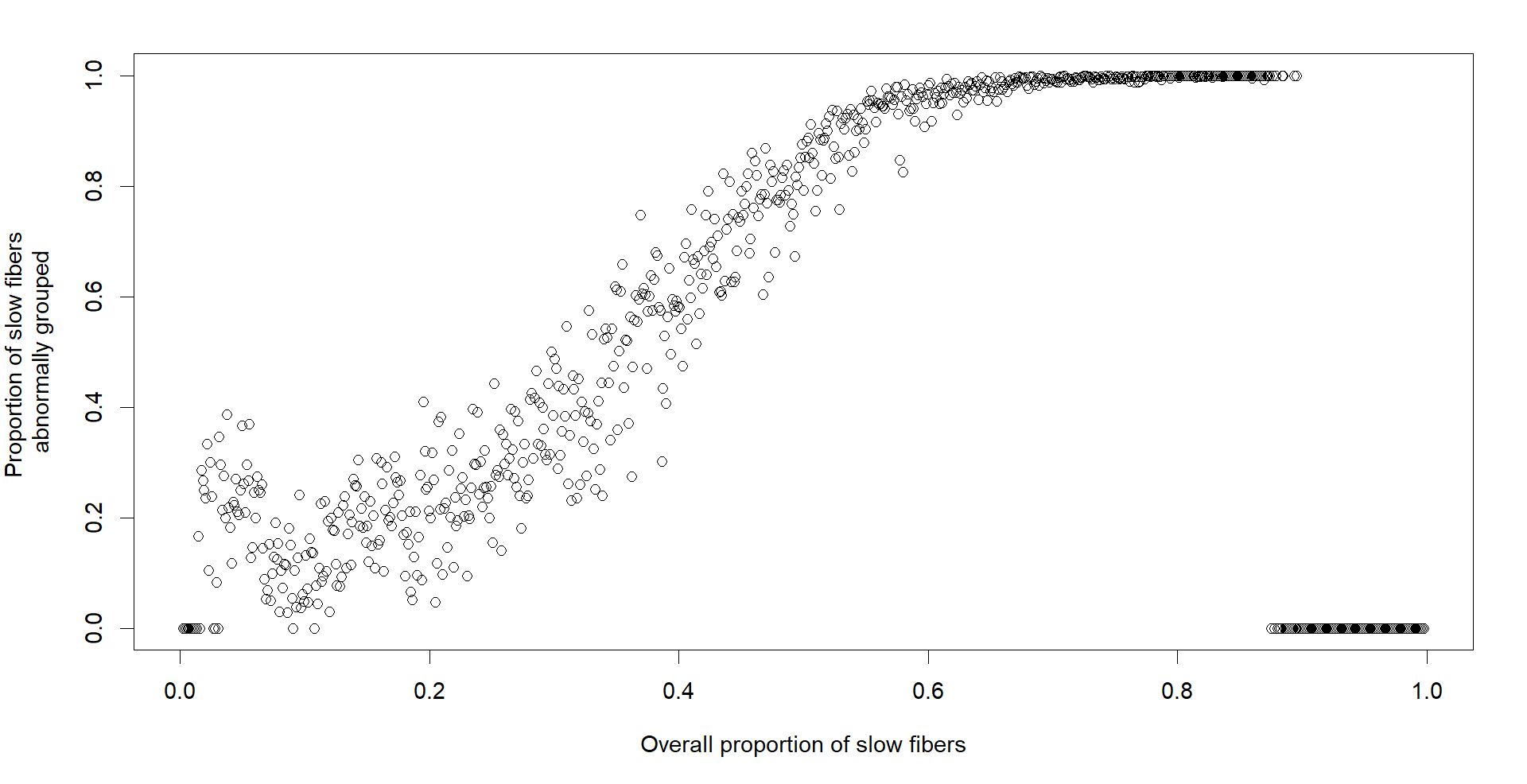


*Figure S1. Proportions of abnormally grouped type I fibers for simulated data with randomly allocated fiber types*

1. *Fiber type plots*


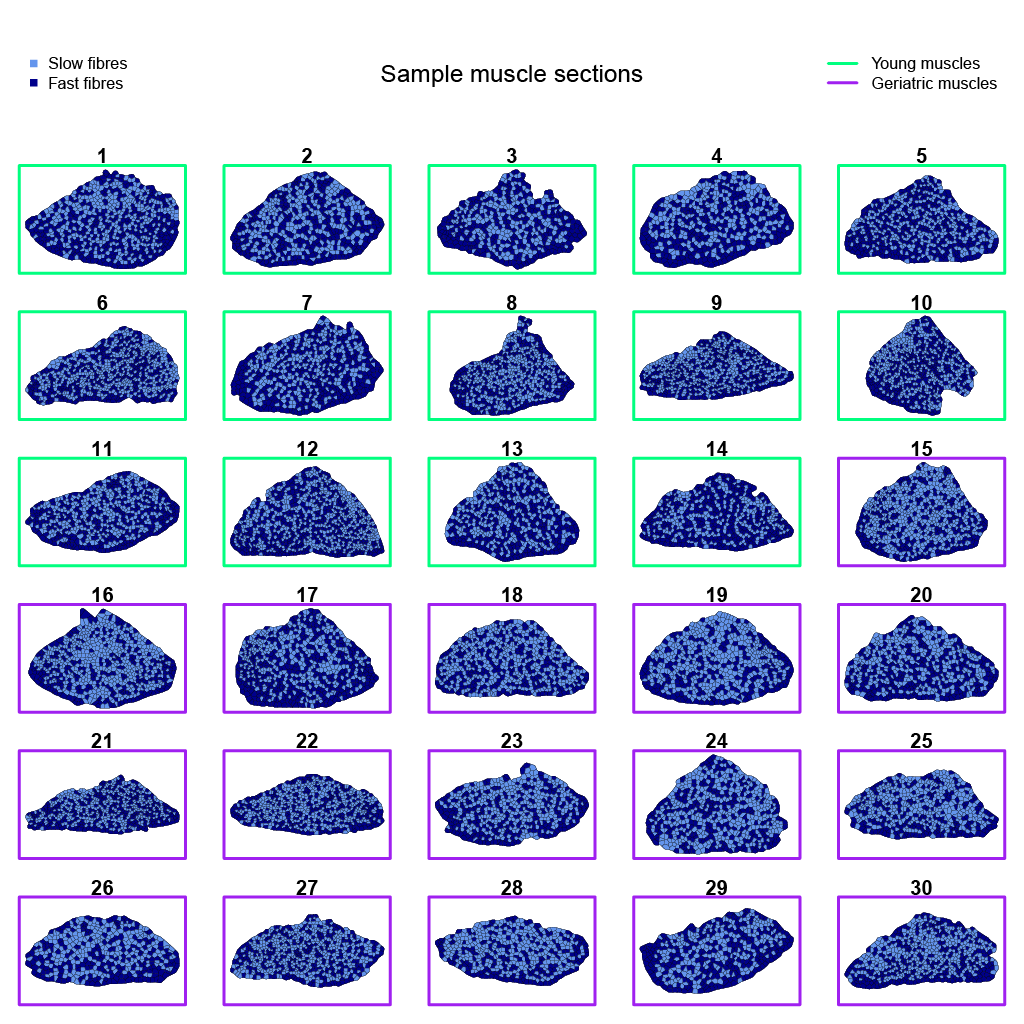


*Figure S2. Plots of fiber types for 30 mouse soleus muscles, 14 young and 16 geriatric*

1. Edelsbrunner, H., D. Kirkpatrick, and R. Seidel. 1983. “On the Shape of a Set of Points in the Plane.” *IEEE Transactions on Information Theory* 29 (4): 551–59.
2. Davies, T. M., M. R. Schofield, J. Cornwall, and P. W. Sheard. 2019. “Modelling Multilevel Spatial Behaviour in Binary-Mark Muscle Fibre Configurations.” *Annals of Applied Statistics* 13 (3): 1329–47.

1. Okabe, A., B. Boots, K. Sugihara, and S. N. Chiu. 2009. *Spatial Tessellations: Concepts and Applications of Voronoi Diagrams*. John Wiley & Sons.
2. Wood, S. N. 2017. *Generalized Additive Models: An Introduction with R*. CRC press.
3. Kelly, N. A, K. G Hammond, M. J. Stec, C. S. Bickel, S. T. Windham, S. C. Tuggle, and M. M. Bamman. 2018. “Quantification and Characterization of Grouped Type i Myofibers in Human Aging.” *Muscle & Nerve* 57 (1): E52–59.
